# Analysis of propeller-induced ground vortices by particle image velocimetry

**DOI:** 10.1007/s12650-017-0439-1

**Published:** 2017-07-10

**Authors:** Y. Yang, A. Sciacchitano, L. L. M. Veldhuis, G. Eitelberg

**Affiliations:** Delft University of Technology, Delft, 2629HS Netherlands

**Keywords:** Propeller, Ground vortices, Particle image velocimetry, Non-uniform inflow

## Abstract

**Abstract:**

The interaction between a propeller and its self-induced vortices originating on the ground is investigated in a scaled experiment. The velocity distribution in the flow field in two different planes containing the self-induced vortices is measured by particle image velocimetry (PIV). These planes are a wall–parallel plane in close proximity to the ground and a wall–normal plane just upstream of the propeller. Based on the visualization of the flow field in these two planes, the occurrence of ground vortices and its domain boundary are analysed. The elevation of the propeller from the ground and the thrust of the propeller are two parameters that determine the occurrence of ground vortices. The main features of the propeller inflow in the presence of the ground vortices are highlighted. Moreover, the analysis of the non-uniform inflow in the azimuthal direction shows that with increasing the propeller thrust coefficient and decreasing the elevation of the propeller above the ground, the variation of the inflow angle of the blade increases.

**Graphical Abstract:**

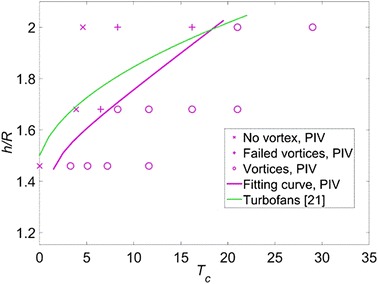

## Introduction

**Fig. 1:**
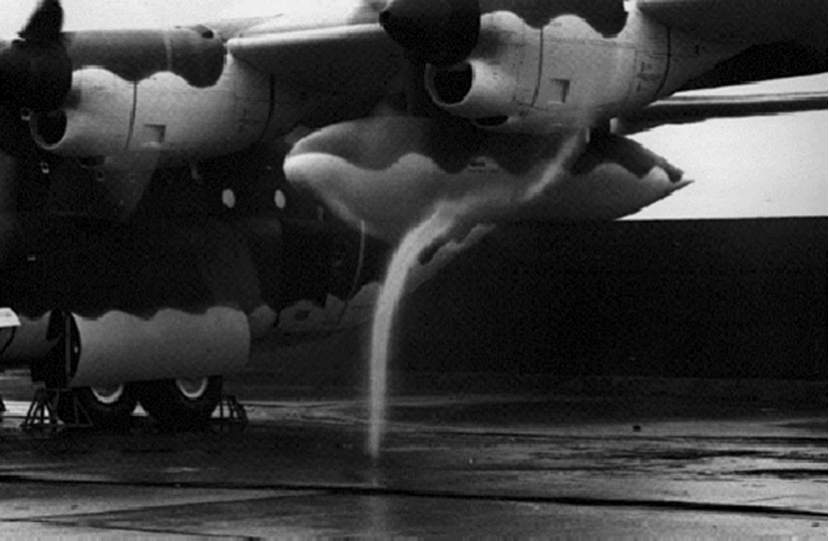
Occurrence of a ground vortex on the outboard propeller of aircraft C130 (Campbell and Chambers 1994) (reprinted with permission from J. F. Campbell and J. R. Chambers, “Patterns in the sky: Natural visualization of aircraft flow fields,” NASA Report No. SP-514, 1994. Used with permission of NASA)

The generation of ground vortices is a phenomenon that occurs during aircraft ground operations. This phenomenon consists of a system of vortices formed on the ground that ascend into the aircraft engine, thus causing unsteady inflow effects. Ground vortices can be observed during aircraft taxiing and engine maintenance when rain droplets are present or air condensation occurs in the vortex region, as shown in Fig. 1 (although there is only one major vortex observed in this figure, there may be other weak vortices which are not observable as they are dependent on the visualization methods (Secareanu and Moroianu 2005), or there are multiple vortices at other instants).

The ground vortices were first investigated because of the concern of foreign object damage (FOD) to the turbofan engine (Rodert and Garrett 1955). It is reported that 40% of engine repairs is due to the foreign object damage when the aircraft is operated at the ground (Golesworthy 1961). Research on the subject has shown that the probability of debris from the ground being ingested by the engine increases with the thrust and decreases with the elevation of the propeller above the ground (Rodert and Garrett 1955).

Because FOD is a severe problem for turbofan engines due to the possibility of damaging fan and compressor stages, the majority of research on ground vortices has been conducted on turbofan engines (Rodert and Garrett 1955; Golesworthy 1961; Klein 1959). Suction tube models have also been introduced to simulate the behaviour of those engines (Secareanu and Moroianu 2005; De Siervi et al. 1982; Murphy and MacManus 2011a; Wang and Gursul 2012; Trapp and Girardi 2010; Karlsson and Fuchs 2000). For propellers, FOD effects due to ground vortices are not as severe as in turbofan engines. However, concerning the engine performance due to inflow distortion, the influence of vortices on propellers requires equal attention as for turbofan engines. The non-uniform inflow of a propeller may also cause structural vibration and noise generation (Povinelli et al. 1972).

Previous investigations have greatly improved the understanding of ground vortices. A stagnation point on the ground (or other fixed structure) must be present as a requirement for ground vortices to exist. In this case, the wall-parallel flow converges in a manner that is similar to that of a sink near the ground (Klein 1959). Vortex shedding from the shroud of the engine was proposed to be the ground vortex origin at crosswind conditions (De Siervi et al. 1982). Intensification of the vorticity of the far-field boundary layer has been proposed as the mechanism for the generation of ground vortices under headwind conditions (De Siervi et al. 1982; Bissinger and Braun 1974; Murphy et al. 2010) for a turbofan engine. In both the headwind and crosswind conditions, the vorticity source is located on the wall (ground or nacelle) and in the far-field boundary layer. By setting different boundary conditions for the ground and nacelle, the no-slip wall boundary condition is found to be necessary for the production of vorticity (Trapp and Girardi 2012). This finding is consistent with the vorticity production equation first formulated by Lighthill, which shows that the vorticity is produced due to the pressure gradient on the no-slip wall (Lighthill 1963).

The vortices formed near the ground are transported to the engine due to the suction. The entry positions of the vortices are at the bottom of the intake around $0.9 R$ tested at a thrust coefficient of $T_{c} = 15$ [corresponding to $U_{\text{in}} /U_{\infty } = 6.25$ (De Siervi et al. 1982)]. The thrust coefficient $T_{c}$ is defined in Eq. (1) for propellers, and $U_{\text{in}}$ is the inflow velocity of the suction tube or the intake of the turbofan:

1 $$T_{c} = T/ \rho U_{\infty }^{2} D^{2} ,$$

where $T$ is the propeller thrust, $\rho$ is the air density, $U_{\infty }$ is the free-stream velocity, and $D$ is the diameter of the propeller.

The ground vortices yield a flow asymmetry that affects the inflow of the engine (Murphy et al. 2010). In this case, the total pressure distortion was shown to increase monotonically with the thrust coefficient when the wind tunnel wall was synchronized with the free-stream velocity (the wind tunnel wall was replaced by a moving belt) to eliminate the boundary layer on the ground (Murphy et al. 2010).

Based on the understanding of the formation mechanism of ground vortices, attempts have been made to decrease or eliminate the influence of ground vortices on turbofan engines. One method that was proposed is the injection of high-pressure air to the origins or paths of ground vortices. A detailed review on this topic is reported in (Trapp and Girardi 2010). Another method is to open the throttle of the engine progressively as the aircraft accelerates, so as to keep the thrust coefficient of the engine at a low value (Glenny 1971).

Although ground vortices which are induced by turbofans have been extensively studied, the flow behaviour of ground vortices induced by propellers has not been investigated yet. Therefore, the objectives of the current research regarding ground vortices induced by propellers are as follows:

1. Build a domain boundary of the occurrence of ground vortices.
2. Gain insight into the propeller inflow due to the interaction between the propeller and ground vortices.
3. Investigate the impact of ground vortices on the propeller performance.

The interaction between the propeller and ground vortices is studied at different thrust coefficients and propeller elevations above the ground. The experimental setup including the propeller model in the wind tunnel and the PIV arrangements is described in Sect. 2. The definitions describing propellers are elaborated in Sect. 3. The domain boundary of the occurrence of ground vortices is investigated in Sect. 4. The impact of ground vortices on the propeller inflow and propeller loadings is analysed in Sect. 5.

## Experimental setup and uncertainty analyses

### Wind tunnel and propeller rigs

Tests were carried out in a low-speed, closed-loop open-jet wind tunnel in the Delft University of Technology. The tunnel denoted as OJF has an octagonal test section, with a height and width of $2.85\;{\text{m}}\; \times \;2.85\;{\text{m}}$ ($18 R\; \times \;18 R$, where *R* is the propeller radius).

The inflow velocity was set at a relatively low value of 2.7 m/s. This speed was chosen to achieve high thrust coefficients needed to generate ground vortices. The boundary layer thickness at the ground table at the streamwise position of the propeller is 0.45 *R*. The turbulence intensity in the free-stream velocity is 0.5%. The details of the measurement and analysis of the boundary layer were reported in (Yang et al. 2016).

**Fig. 2:**
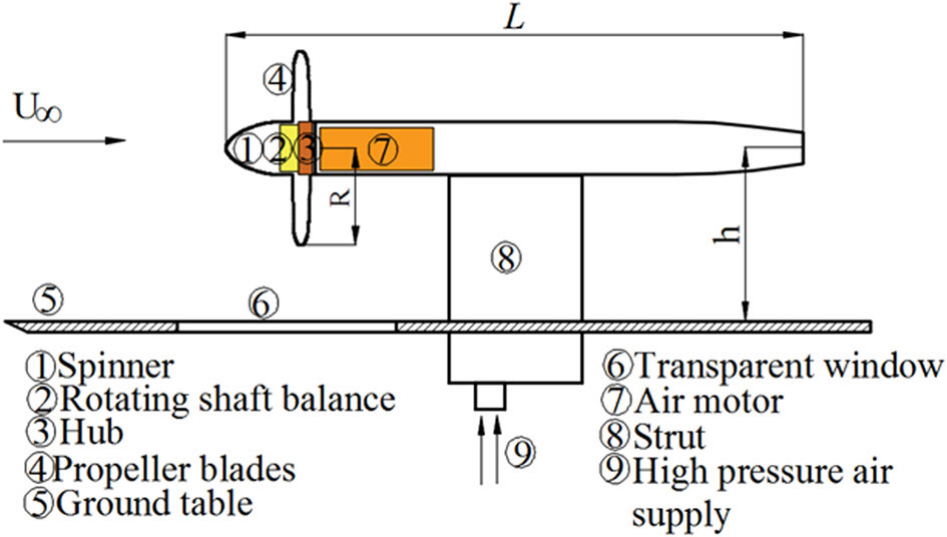
Schematic overview of the experimental arrangement

The propeller is driven by an air motor, which is represented by part 7 in Fig. 2. The maximum power of the engine is 98 HP (73.09 KW) when operated at air supply (part 9 in Fig. 2) of 34.47 Bar and mass flow of 0.907 kg/s. The propeller is directly coupled to a rotating shaft balance (*RSB*, part 2 in Fig. 2) that measures the isolated thrust and torque produced by the propeller. The range of the balance is $\pm 350 {\text{N}}$ for the axial force and $\pm 30 {\text{Nm}}$ for the axial torque.

**Fig. 3:**
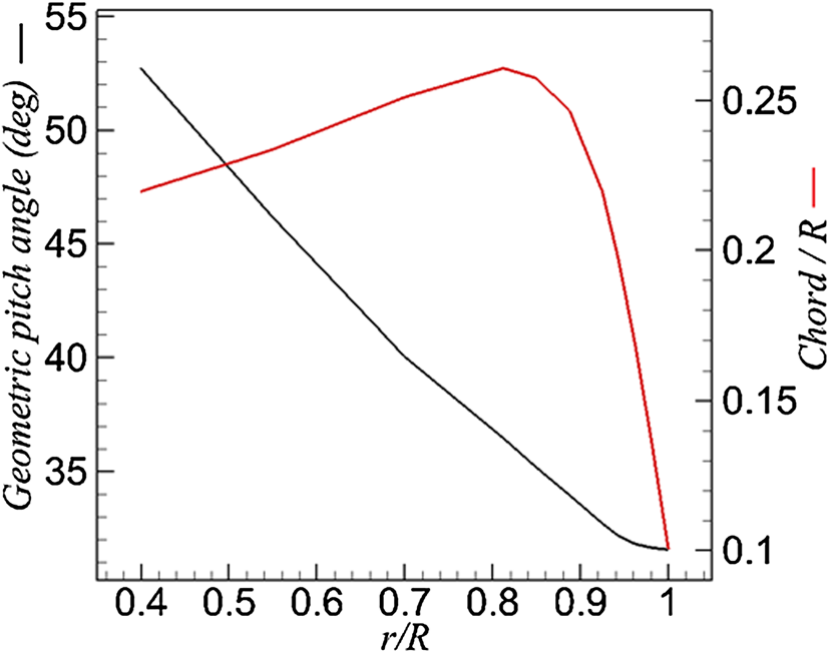
Distributions of the geometric pitch angle and chord length along the radial direction of the blade

The isolated eight-bladed scale model is a Fokker F29 propeller, with the radius *R* = 0.152 m and the total length of the propeller model *L* = 0.904 m, involving spinner, hub (with diameter of 0.084 m) and nacelle. The blade geometric pitch angle $\beta$ (defined in Fig. 5) varies from $53^{^\circ }$ to $32^{^\circ }$ from the root to the tip of the blade as shown by the black curve in Fig. 3. The geometric pitch angle of the blade is set to $39^{^\circ }$ at 0.75 *R* radial position, which corresponds to a typical high loading condition. The chord length distribution along the radial direction is shown by the red curve in Fig. 3, and the chord length at 0.75 radial position is 0.25 *R*.

The maximum rotating speed of the propeller in our test is 5000 rpm, which corresponds to a Mach number at the ¾ radial position of 0.18, and a Reynolds number of 147,000. Because the propeller is designed for the Fokker F29 conceptual aircraft, the scaling effects compared to the full-scale propeller cannot be provided. However, an estimate can be made by considering a similar aircraft of Fokker F27 (with engine PW127B of rotating speed 1200 rpm). This corresponds to a tip Mach number of approximately 0.67. It can be found that the Mach number of the scaled model is lower than the real propeller. Although the chord length of the blade of Fokker F27 is not available for us, the size of the real propeller is definitely several times of our model; the tip speed was already shown to be 3.7 times as high as our model. Therefore, the Reynolds number of the real propeller is approximately one order of magnitude higher than our model.

Although the Mach number and the Reynolds number are not achieved to be the same as the real situation, the non-dimensional parameters, e.g., the advance ratio *J* and thrust coefficient $T_{c}$, are set at realistic values in our measurements. The advance ratio is defined as follows:

2 $$J = U_{\infty } / nD ,$$

where $n$ is the rotating speed of the propeller with unit of round/second.

To simulate a propeller engine operating near the ground, a flat table is positioned under the propeller, as shown in Fig. 2. The width of the ground table is $18 R$, and the diameter of the inflow stream tube of the propeller is around $2.4 R$ at $T_{c}$  = 11.6. Hence, the width of the table is enough to avoid any influence from the table edges. The distance from the leading edge of the table to the projection of the blade leading edge on the ground is $6 R$. The propeller suction induces a low-pressure region upstream of the propeller on the ground; the pressure minimum appears at around $1 R$ upstream the propeller (Yang et al. 2014). From the reported pressure measurements (Yang et al. 2014), it can be concluded that the propeller influence on the table leading edge is negligible. The ground table has a transparent window insert, which allows optical access for PIV cameras.

Previous research on turbofans suggests that the occurrence of ground vortices is determined by two parameters: the height ratio of the propulsor ($h/R$, $h$ is defined in Fig. 2), and the inlet velocity ratio ($U_{\text{in}} /U_{\infty }$) (Nakayama and Jones 1996). The inlet velocity ratio of turbofans is replaced by the advance ratio of propellers in our research, which was defined in Eq. (2). The test matrix is shown in Table 1. Whilst maintaining a constant wind tunnel free-stream velocity, the propeller influence on the occurrence of ground vortices and the influence of the ground vortices on the propeller are studied at different advance ratios and elevations of the propeller above the ground. The advance ratio of the propeller is adjusted by changing the rotational speed of the propeller.

**Table 1:** Test matrix of the experiments

$U_{\infty }$ ($m/s$)	Height of the propeller from the ground, $h$	Rotating speed of the propeller, rpm	Advance ratio of the propeller, $J$	*Re* of the blade cross section at the ¾ radial position
2.7	$1.46 R$	5000, 4500, 3500, 2800, 2500, 2100, 1900, 1500, 1300	0.11–0.41	147,000–38,000
2.7	$1.67 R$	3500, 3000, 2500, 2100, 1800, 1400	0.15–0.38	103,000–41,000
2.7	$2.00 R$	4000, 3500, 3000, 2100, 1600	0.13–0.33	118,000–47,000
2.7	$3.00 R$	4500, 4000, 3500, 3000, 2500, 2100	0.12–0.25	133,000–62,000

### PIV setup

Planar PIV measurements were conducted at the wall-parallel plane, whilst stereoscopic-PIV tests were carried out at the wall-normal plane due to the strong out-of-plane component of the velocity. The measurement system was composed by two LaVision Imager Pro LX 16 M cameras (CCD sensor of 4870 pixels × 3246 pixels, 12 bit resolution, 7.4 µm pixel pitch) and a Quantel Evergreen 200 laser (dual pulsed Nd:YAG laser, 200 mJ energy per pulse). The laser sheet thickness was 2 mm. The flow was seeded with micron-sized water–glycol particles produced by a SAFEX Twin Fog Double Power smoke generator inserted in the settling chamber. The median seed particle was 1 μ, according to the manufacturer’s specifications. PIV measurements in this paper are not synchronized with the propeller rotation.

**Fig. 4:**
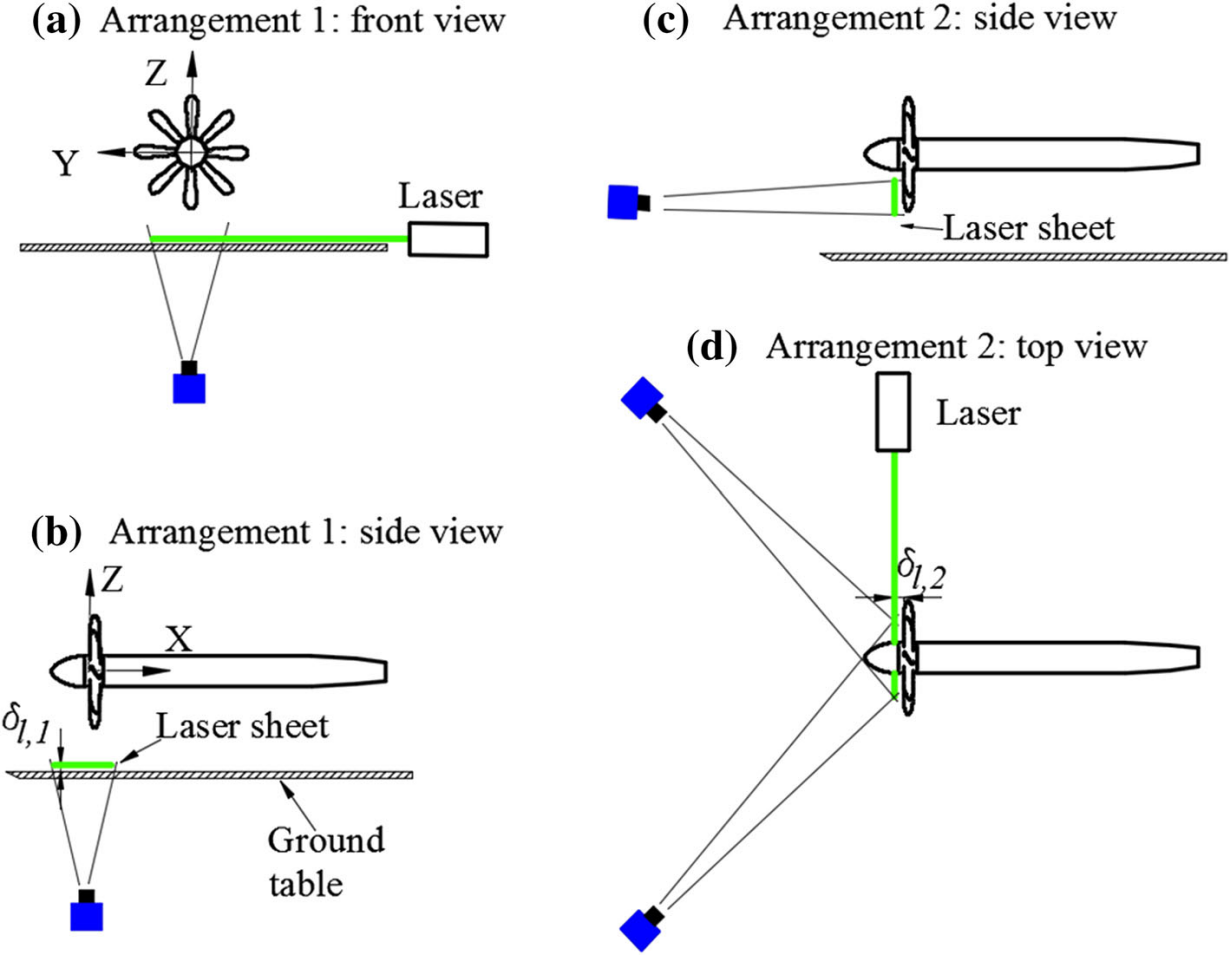
PIV arrangements. *Left* PIV arrangement for the measurement in the wall-parallel plane (arrangement 1); *right* PIV arrangement for the measurement in the plane upstream of propeller (arrangement 2)

For PIV arrangement 1, measurements are conducted at $\delta_{l,1}$  = 0.046 *R*, which is 7 mm above the ground, as shown in Fig. 4a and b. The imaging system is based on a 35 mm Nikkor objective set at $f\# \cdot \cdot 4$ and the magnification factor is 0.0735. The processing is conducted with interrogation window size 128 pixels × 128 pixels, 75% overlap, Gaussian-weighting function, and the vector pitch is 3.22 mm (0.0212 *R*). For the second PIV arrangement, measurements are carried out at $\delta_{l,2} = 0.08 R$, which is 12 mm upstream of the leading edge of the propeller blade, as shown in Fig. 4c and d. In this case, the two cameras are positioned with 45° view angles, one in forward scatter and the other in backward scatter. The forward and backward scattering cameras are based on 200 mm Nikkor objectives set at $f\# \cdot \cdot 5. 6$ and $f\# \cdot \cdot 4$, respectively. To minimize the reflection from the blades of PIV arrangement 2, the laser is projected from the top of the figure, as shown in Fig. 4d. The magnification factor is 0.1076. The processing is conducted with interrogation window size of 128 pixels × 128 pixels, 75% overlap, Gaussian-weighting function, and the vector pitch is 2.21 mm (0.0145 *R*). In both arrangements, the number of images pairs recorded is 250 per testing condition. Due to inhomogeneous distribution of the seed particles and non-uniform laser light illumination, a relatively large interrogation window ($128 \times 128$ pixels) was chosen to ensure the reliability of the results.

### Uncertainty analyses

The uncertainty of PIV data is estimated by the image matching method (Sciacchitano et al. 2013). The image matching method uses the measured velocity field to match the particle images of the recordings based on the processing algorithm (for example, by window deformation or window shift). The approach detects particle images in each interrogation window. In case of exact velocity measurements, the particle images of the two recordings would match perfectly. In real experiments, the paired particle images do not match exactly and feature a positional disparity between them. The positional disparity is computed as the distance between the centroids of the particle images. The measurement uncertainty is determined within each interrogation window from the mean value and the statistical dispersion of the positional disparity vector.

In the wall-parallel plane, the uncertainty of the instantaneous velocity fields at 95% confidence level is 0.02 m/s. For the wall-normal plane PIV measurement, the uncertainty is 0.18 m/s for the in-plane velocity components and 0.17 m/s for the out-of-plane velocity component. The uncertainty of the time-averaged flow field is 0.01 m/s in the wall-normal plane. For the error analyses of the PIV measurements as shown above from the conventional methods, it should be mentioned that they are underestimated. First, the interrogation window size for the PIV measurements of ground vortices is as big as 128 × 128 *pixels*. Although the conventional methods correctly get the random part of the error, the systematic error due to the averaging over a large region is underestimated. In addition, some error sources are not accounted for in the current analysis, e.g., the particle velocity lag in the vortex centre, where the swirl velocity is high. Furthermore, there is also an issue of high particle density and low intensity of the laser light (a large field of view is required during the tests) during our measurements, which introduces problems of multi-scattering and leads to inhomogeneities of the particle density.

Repeated measurements for the wind tunnel free-stream velocity and propeller thrust were conducted to calculate the uncertainties. The uncertainty of the free-stream velocity is 0.3% at 2.7 m/s. The uncertainty of the thrust measurement is 0.27 N for a typical median thrust of 14.24 N, yielding a relative error of 1.9%.

## Definitions

**Fig. 5:**
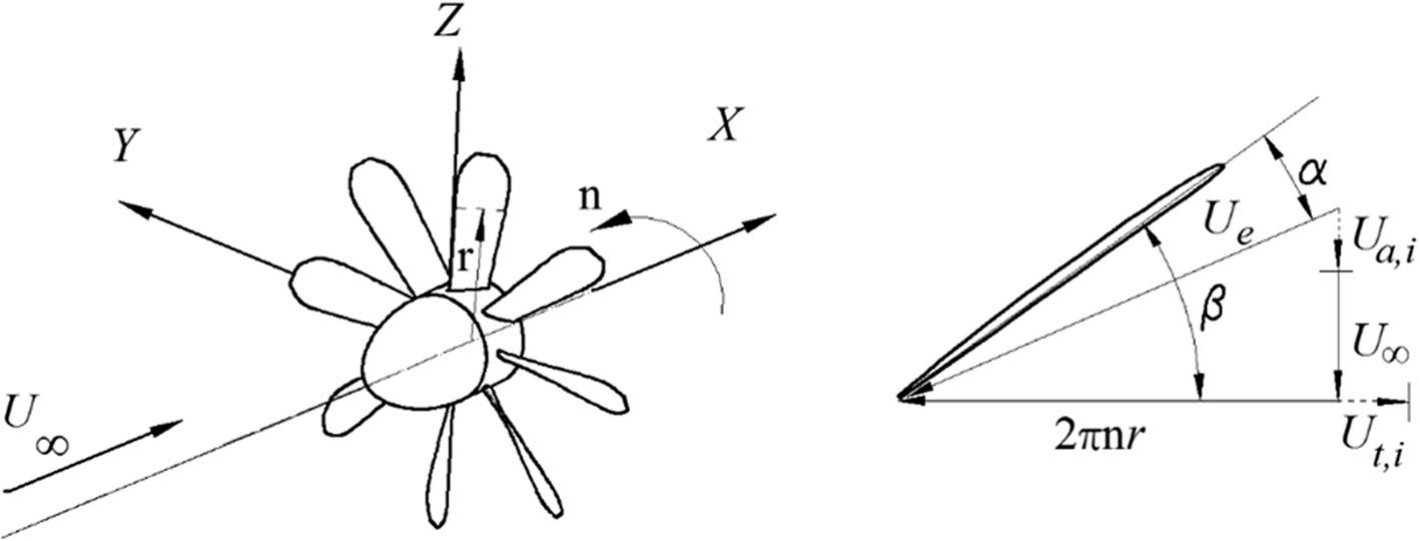
*Left* Definition of the coordinate system of the propeller; *right* definition of the incidence angle of the cross section of the blade at the radial position $r$

The interaction between the propeller inflow and the ground yields induced velocity components in both the axial and azimuthal directions. As a result, the propeller inflow field is distorted and has strong gradients in the azimuthal direction (Wang and Gursul 2012). The induced velocity components yield a variation of the blade incidence angle and dynamic pressure, thus causing a change in the blade loads. The axial and tangential velocities of the propeller are defined as

3 $$U_{a0} = U_{\infty } \left( {r,\psi } \right) + U_{a,i} \left( {r,\psi } \right) ,$$

4 $$U_{t0} = 2\pi nr + U_{t,i} \left( {r,\psi } \right) ,$$

where $U_{a,i}$ is the induced axial velocity, *n* is the propeller angular speed, and $U_{t,i}$ is the induced tangential velocity. All these parameters are illustrated in Fig. 5.

The blade incidence angle is defined as

5 $$\alpha \left( {r, \psi } \right) = \beta - { \tan }^{ - 1} \left( {U_{a0} /U_{t0} } \right) ,$$

where *β* is the blade geometric pitch angle. The blade incidence angle is utilized to analyse the vortex impact on the propeller in Sect. 5.

Besides the thrust coefficient defined in Eq. (1), another important characteristic of the propeller is the torque coefficient which is defined as

6 $$Q_{C} = \frac{Q}{{\rho U_{\infty }^{2} D^{3} }},$$

where $Q$ is the torque measured at the shaft. The efficiency of the propeller represents the work in the forward direction of the propeller divided by the shaft power:

7 $$\eta = \frac{{TU_{\infty } }}{Q2\pi n} = \frac{{T_{C} }}{{2\pi Q_{C} }}J .$$

For a propeller with an axisymmetric inflow, the axial component of the time-averaged velocity in the propeller inflow can be predicted by the actuator disk model (Yang et al. 2012), which is shown as below

8 $$U_{\text{eq}} = U_{\infty } \sqrt {1 + 8 \cdot T_{C} /\pi } ,$$

where $T_{c}$ was defined Eq. (1) already. This equivalent velocity is determined by the free-stream velocity and the thrust generated by the propeller, which together determine the generation of ground vortices. This velocity is utilized to normalize the velocity and vorticity of PIV measurement results.

## Measurement results

### Instantaneous flow field in the wall-parallel plane

Examples of typical instantaneous flow fields formed on the wall-parallel plane are discussed in this section. The plane near the wall (the plane is $\delta_{l} =$ 7 mm above the ground, and $\delta_{l} /R = \varvec{ }$ 0.046) is selected so as to capture the flow field near the origin of ground vortices.

**Fig. 6:**
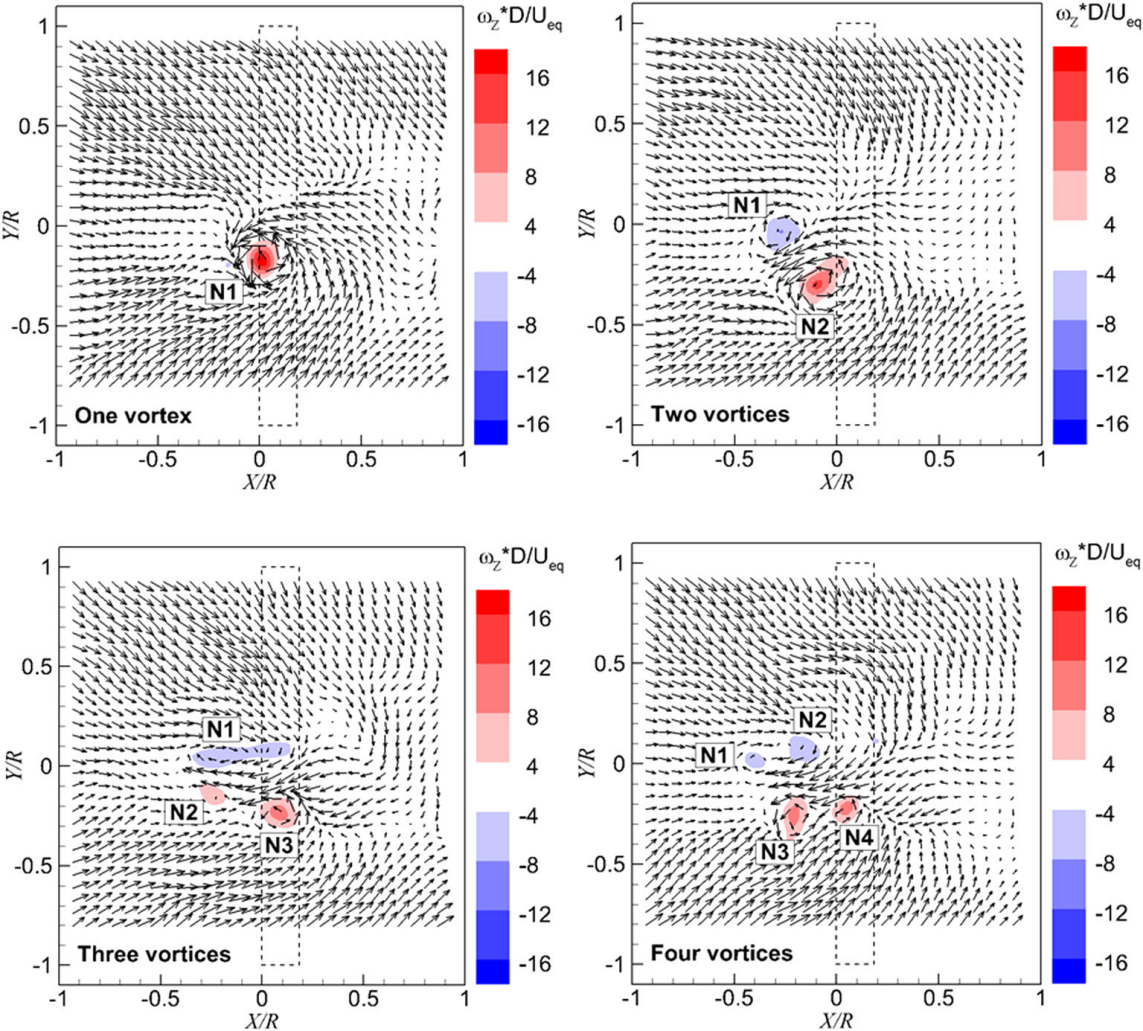
Typical instantaneous velocity fields in the *horizontal* plane above the ground, every third measured vector is shown. Superimposed are the color-coded magnitudes of non-dimensional wall-perpendicular vorticity. $T_{c}$  = 11.6, *h/R* = 1.46. ‘*N*’ represents the topological feature of a node. The *dashed line* indicates the propeller projection

**Fig. 7:**
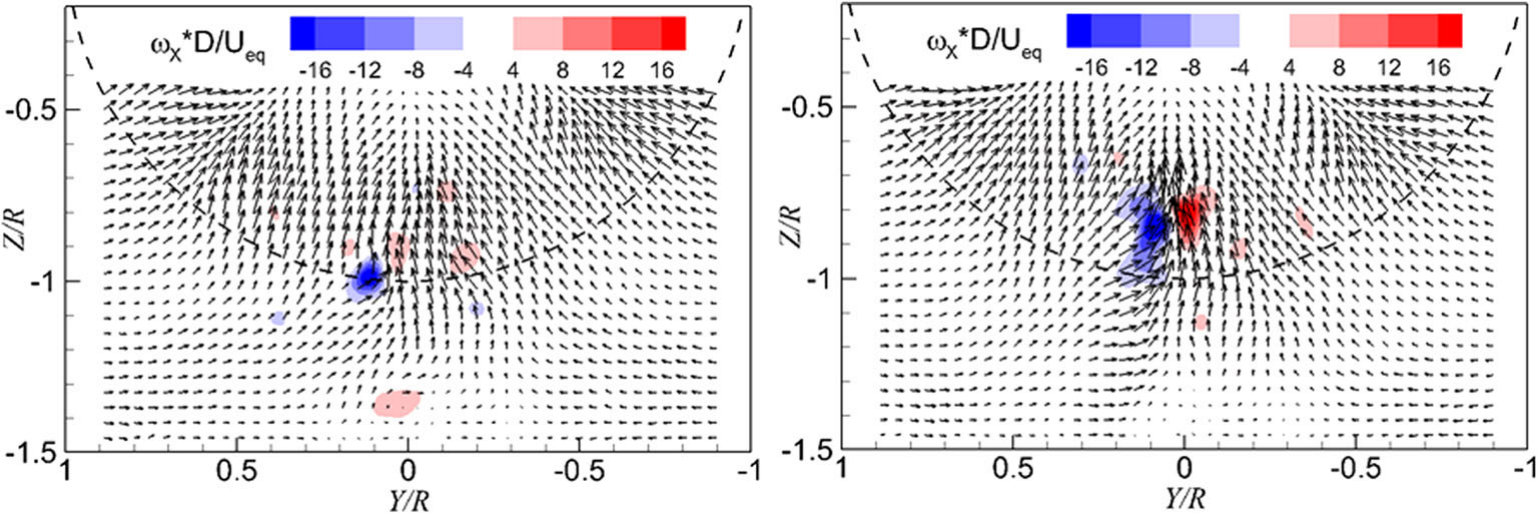
Instantaneous flow fields in the wall-normal plane. *Left* one dominant vortex; *right* two dominant vortices. $T_{c}$  = 11.6, *h/R* = 1.46

The location of vortices is identified by the local maximum of vorticity magnitude. There is one peak of vorticity magnitude shown on the top left of Fig. 6; therefore, the flow field is interpreted as representing one dominant ground vortex. In a similar manner, the flow field in the top right of Fig. 6 is interpreted as having two dominant ground vortices. Flow fields with three and four dominant vortices are also observed, as shown in the bottom row of Fig. 6. The sign of the Z-component vorticity is defined as positive when it has the same direction as the Z axis as that defined in Fig. 5 and the same for the sign of X-component vorticity, as shown in Fig. 7.

### Flow field in the wall-normal plane

The instantaneous flow fields in the wall-normal plane just upstream of the propeller are presented in Fig. 7, where the color-coded contour plot of the axial component of the vorticity is superimposed on the velocity vector field. The vortices entering the propeller can be identified by the peak value of the vorticity. There is one dominant vortex shown on the left-hand side of Fig. 7, whereas there are two dominant vortices on the right-hand side of Fig. 7.

## Data analysis

### Domain boundary of the occurrence of ground vortices

**Fig. 8:**
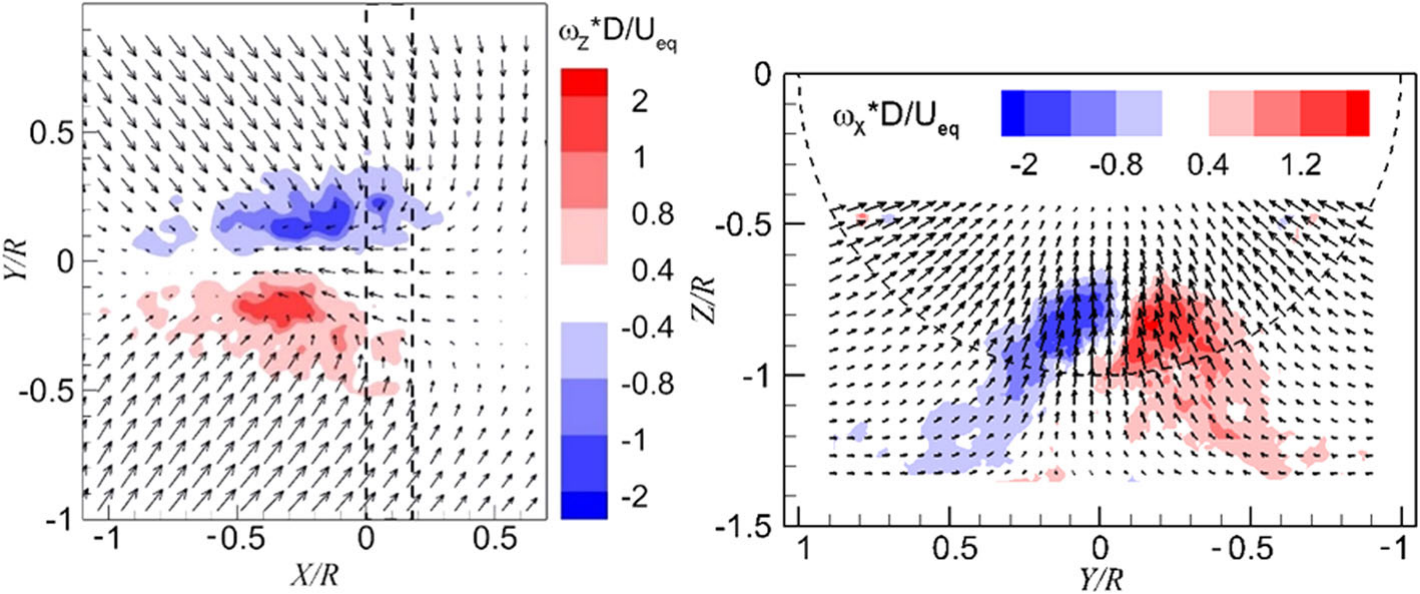
Distributions of vorticity in the time-averaged flow field. *Left* wall-parallel plane; *right* wall-normal plane directly upstream of the propeller. $T_{c}$  = 27.3, *h/R* = 1.46

The determination method for the occurrence of vortices is by detecting the concentrated vorticity region in the time-averaged flow field. The concentrated vorticity in the wall-parallel plane and the wall-normal plane is shown in the left- and right-hand sides of Fig. 8, respectively. The time-averaged flow fields show a pair of vortices. In addition, the vorticity in the time-averaged flow field (in the range of −2 to 2) is an order of magnitude lower than the instantaneous flow field (in the range of −20 to 20), which is due to the smearing effect.

A parameter is defined here to evaluate the concentration of vorticity, $C_{\text{vort}}$, which is the ratio between the maximum magnitude of the vorticity in the time-averaged flow field, $\left| \omega \right|_{ \hbox{max} }$, and the vorticity magnitude in the region assumed to be unaffected by the ground vortices, $\left| \omega \right|_{\text{unaff}}$:

9 $$C_{\text{vort}} = \frac{{\left| \omega \right|_{\hbox{max} } }}{{\left| \omega \right|_{\text{unaff}} }} .$$

These unaffected regions are chosen at a $3 \times 3$ kernel centred at $\left[ {X/R, Y/R} \right] = \left[ { - 1, 0.7} \right]$ in the wall-parallel plane and $\left[ {Y/R,Z/R} \right] = \left[ { - 0.8, - 1} \right]$ in the wall-normal plane. The criterion applied herein is that if the ratio is larger than 10 (this value is determined by considering that the concentrated vorticity should be one order of magnitude larger than the vorticity from turbulence), it is considered to be concentrated vorticity; otherwise, there is no concentrated vorticity in the flow field.

**Fig. 9:**
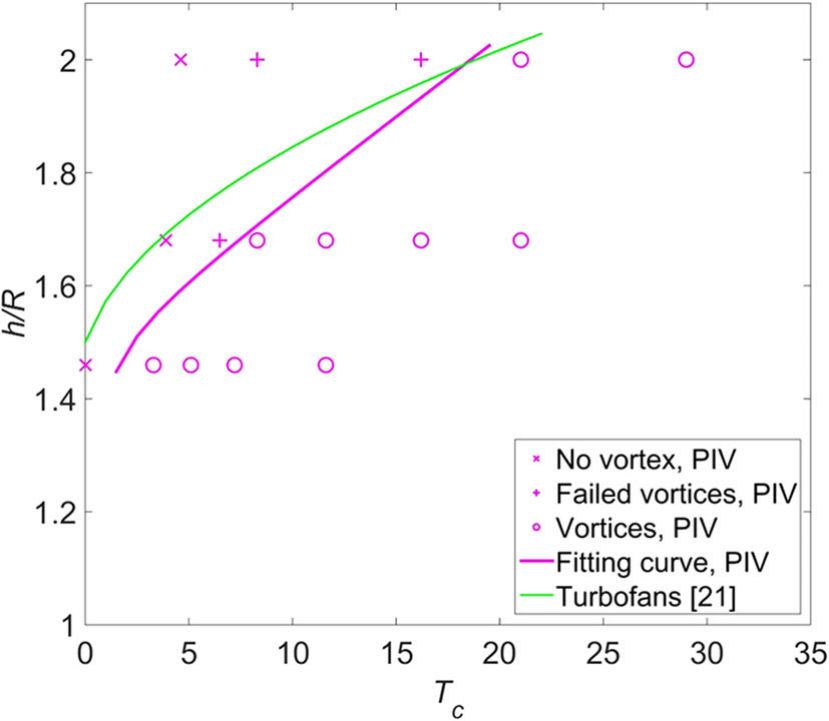
Domain boundary of occurrence of ground vortices induced by the propeller. The data of turbofans (*green curve*) were reported in Nakayama and Jones (1996)

If there is no concentrated vorticity in the flow field either near the ground or upstream of the propeller, it is defined as the case ‘no vortex’. If there is concentrated vorticity in the flow field both near the ground and upstream of the propeller, it is defined as the case ‘vortices entering the propeller (vortices)’. If there is concentrated vorticity in the flow field near the ground but not existing directly upstream of the propeller, it is defined as the case ‘failed vortices’ (these failed vortices are also observed in Wang and Gursul (2012)). A map of ‘no vortex’ (symbol ‘x’), ‘failed vortices’ (symbol ‘+’), and ‘vortices entering propeller’ (symbol ‘o’) is shown in Fig. 9.

The fitting curve by connecting the midpoints between the vortices and failed vortices or no vortex at $h/R = 1.46, 1.67,$ and 2.0 is shown by the solid purple curve in Fig. 9. The fitting curve divides the domain into two sub-domains: the upper left domain represents no vortex entering the propeller, whilst the bottom right domain represents vortices entering the propeller. In other words, as the height ratio decreases and the thrust coefficient increases, the ground vortices occur.

It should be noted that only the parameters of $T_{c}$ and $h/R$ are taken into account when predicting the occurrence of ground vortices, and the parameter of free-stream velocity is kept constant. The vorticity transported from the free-stream velocity is also one source of vorticity to form ground vortices as reported in De Siervi et al. (1982). The free-stream velocity is very likely one factor determining the occurrence of ground vortices. The varying of the free-stream velocity to investigate the domain boundary is ascribed to future work.

In addition, the green curve is the domain boundary of occurrence of ground vortices for turbofans which is reported in Nakayama and Jones (1996); it shows that the ground vortices induced by turbofans occur at lower thrust coefficients than that induced by the propeller for the same height ratio. There is a major difference between a propeller and a turbofan, which is the shroud of a turbofan. For a propeller without a shroud, the vortex system in the slipstream would induce velocity of components both in the tailwind direction and in the crosswind direction near the ground. This additional induced flow would increase the strength of the shear flow near the ground (this shear flow can be found in Fig. 6 near the ground), so as to increase the chance of the generation of ground vortices. However, the results of our tests give a contrary trend, which means the effect of the shroud may not be a dominant factor for the difference between our results and the results of turbofans. This discrepancy of the results between a propeller and a turbofan is perhaps due to the different free-stream velocities, which play a role in the forming of ground vortices (Brix et al. 2000).

### Influence of the thrust coefficient and the height ratio on the propeller inflow

- Non-uniform inflow of the propeller due to the impact of ground vortices.

As observed in Fig. 6, there are topologies of one ground vortex, two ground vortices, and multiple ground vortices ascending from the ground and entering the propeller at different instants. For each instant, the inflow of the propeller, which is the vortex-induced flow superimposed on the free stream and the propeller-induced flow, is different. An analysis of the inflow of the propeller at each instant to analyse the impact of the vortex on the propeller is not performed in this paper; instead, the integral effect of the vortices on the propeller is analysed from the time-averaged flow field upstream of the propeller.

**Fig. 10:**
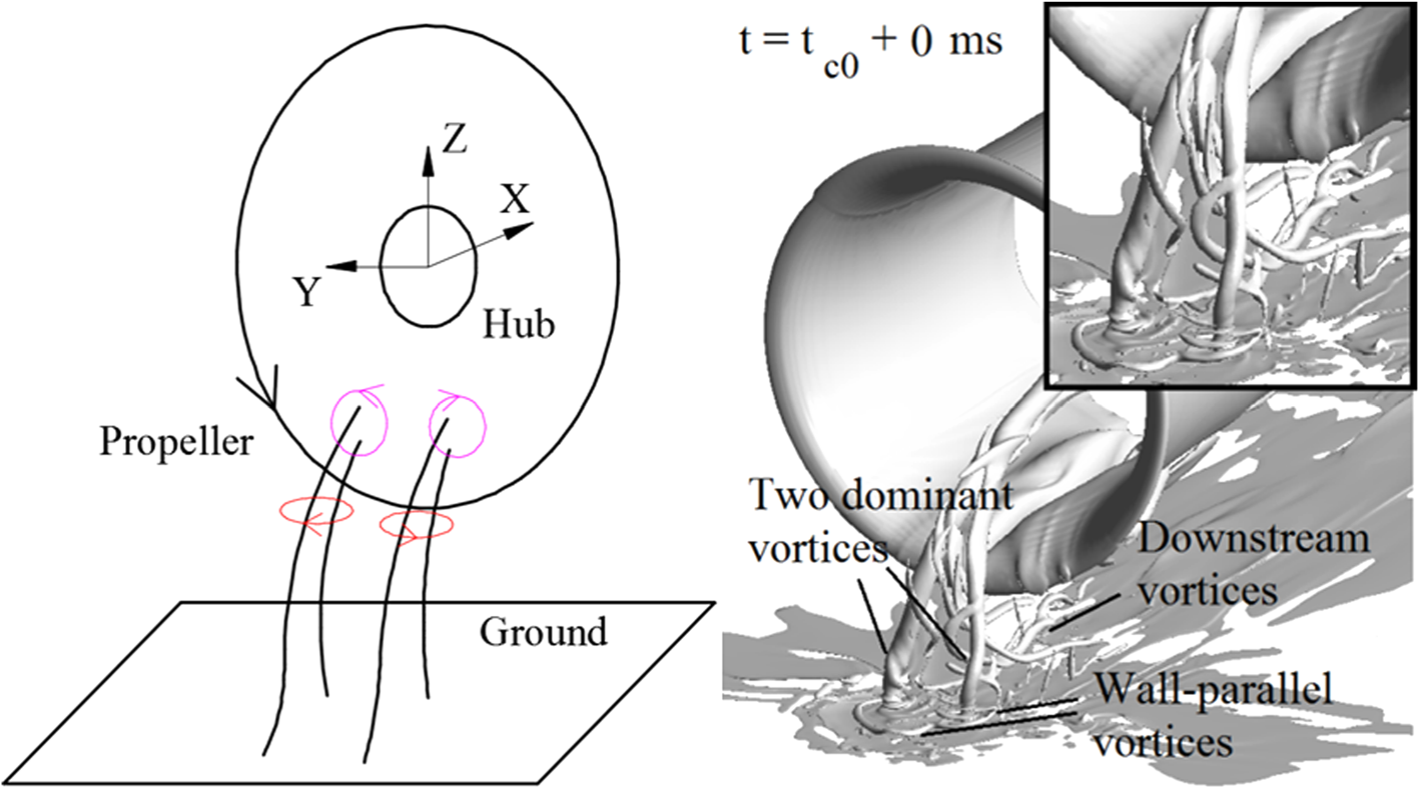
*Left* schematic of ground vortices entering the propeller in the time-averaged flow field; *right* topology of ground vortices induced by the actuator disk model, which is simulated by a CFD analysis and reported in (Yang 2017)

The time-averaged flow fields, as shown in Fig. 8, with contour of the out-of-plane component of the vorticity, feature a pair of vortices both in the wall-parallel plane and in the wall-normal plane. A schematic to represent the topology of the time-averaged flow fields is drawn in the left-hand side of Fig. 10. The vortices enter the propeller in an oblique angle (this is deduced from a 3D flow topology found from a computational fluid dynamics (CFD) simulation by the authors (Yang 2017), as shown on the right-hand side of Fig. 10), the *Z*-component of the vortices is represented by the red circles, and the *X*-component of the vortices is represented by the purple circles.

**Fig. 11:**
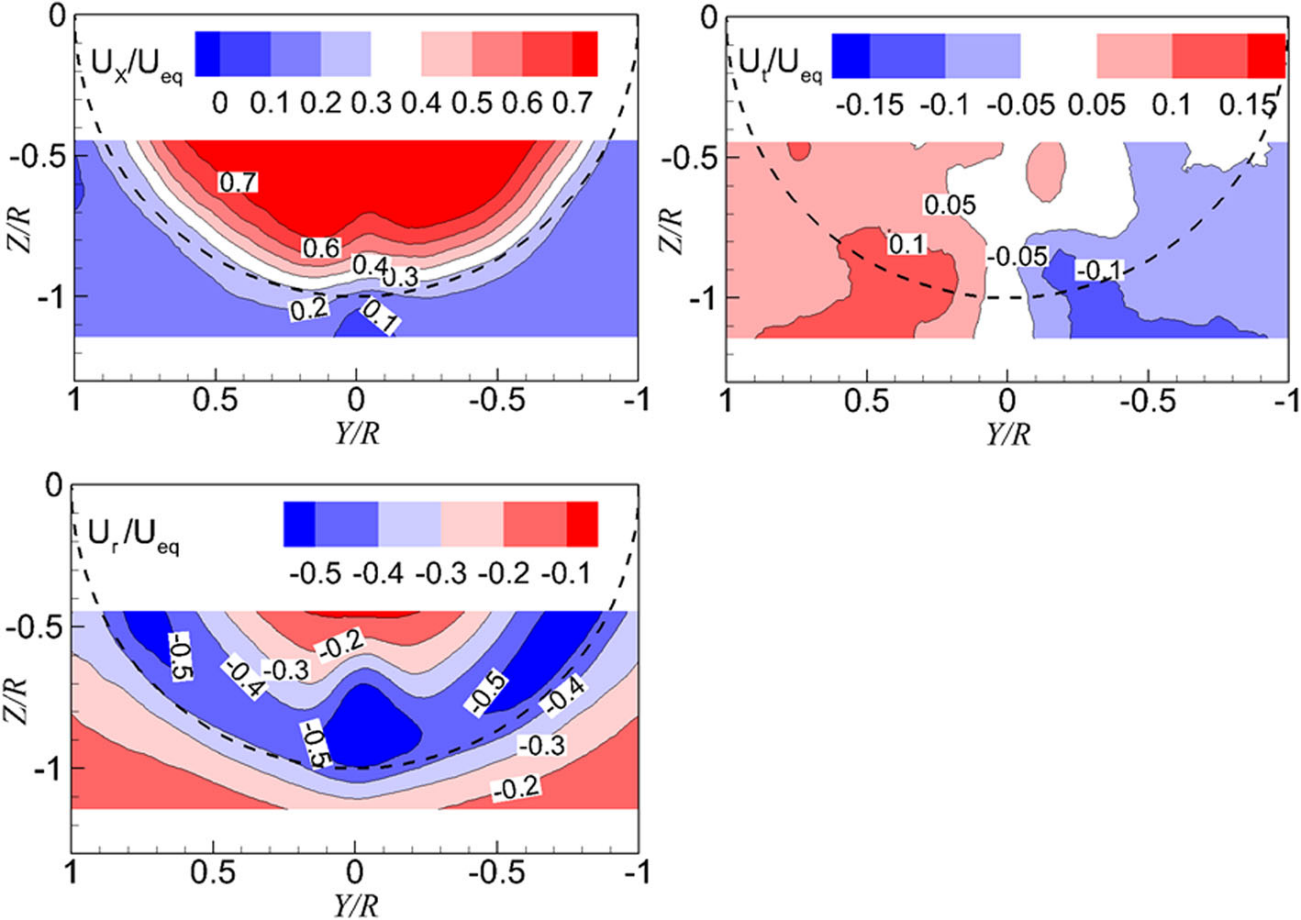
Distribution of the axial (*top left*), the tangential (*top right*), and the radial (*bottom left*) flow velocities in the plane upstream of the propeller. $T_{c} = 42.1$, $h/R = 1.46$

The analysis of the impact of vortices on the propeller inflow is performed at the situation of $T_{c} = 42.1$, and $h/R = 1.46$, which is the case with a strong effect of the vortices on the propeller inflow. The resulting flow fields, due to the interaction between vortices and the propeller, are shown by the distributions of the axial velocity (top left of Fig. 11), the tangential velocity (top right of Fig. 11), and the radial velocity (bottom left of Fig. 11).

**Fig. 12:**
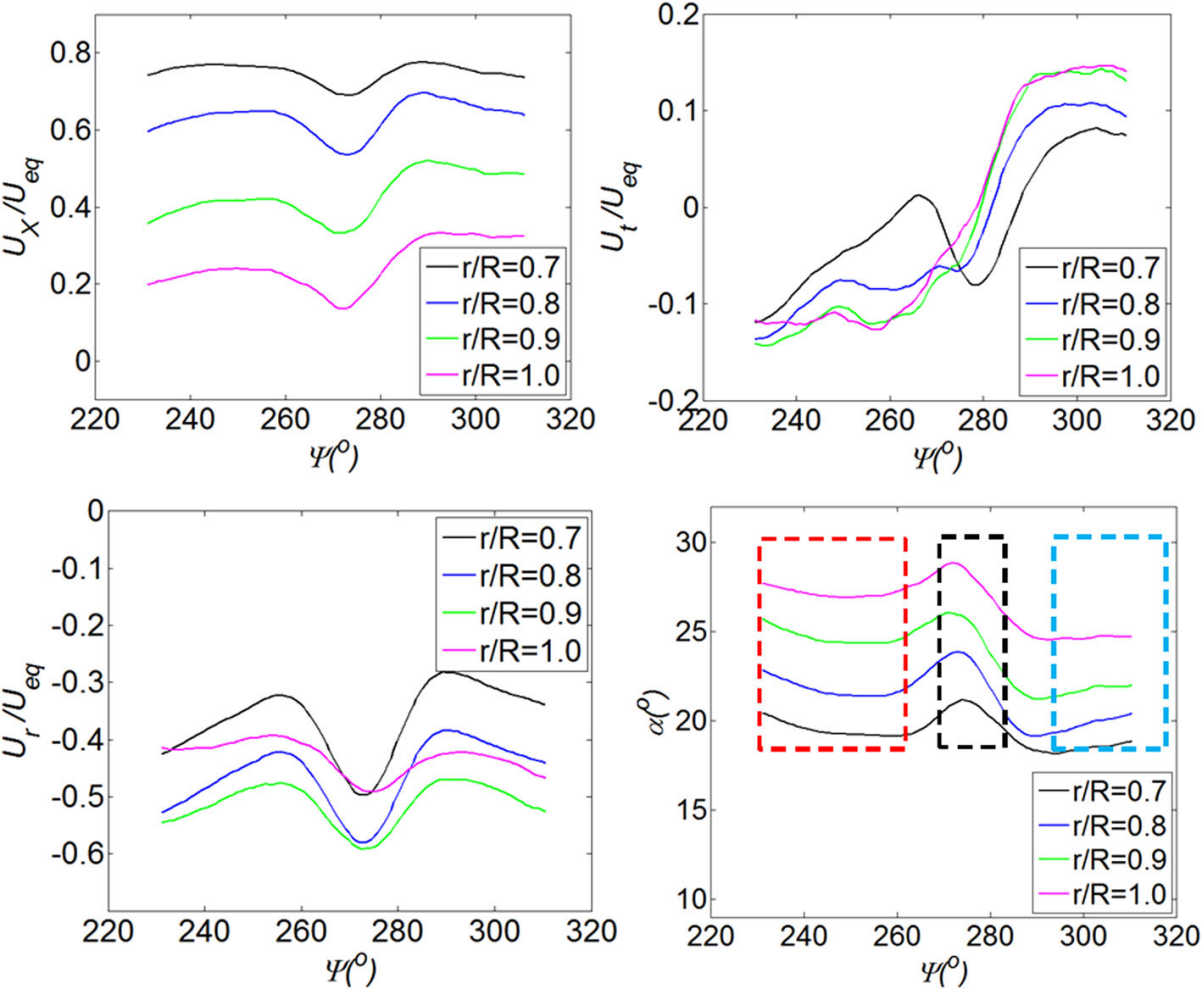
Impact of ground vortices on the propeller inflow. *Top left* distribution of the axial velocity; *top right* distribution of the tangential velocity; *bottom left* distribution of the radial velocity; *bottom right* distribution of the incidence angle of the blade

The analysis of the flow fields, as shown in Fig. 11, is performed together with analysing the distribution of velocities along the circumferential direction. The radial positions chosen for analysis are $r/R = 0.7, 0.8, 0.9, {\text{and}}\, 1.0$, which are inside the region influenced by the ground vortices. The distribution of the axial flow velocity features a region with a dent at the circumferential position around $\varPsi = 270^{\circ}$ and two bulges on the two sides of the dent region, as shown in the top left of Fig. 12. These bulges and dent of the axial velocity are due to the vortex entering the propeller in the wall-normal direction ($\omega_{Z}$) which is represented by the red circles, as shown in Fig. 10.

The distribution of the tangential velocity is shown in the top right of Fig. 12, which features half of the field of view with positive tangential velocity and the rest is negative. This is mainly due to the two vortices entering the propeller in the propeller axial direction (purple circle in Fig. 10). The vortex on the left-hand side has the rotating velocity of the counter clockwise direction, and vice versa for the vortex on the right-hand side. The entering position of the vortex is approximately at the radial position of $r/R = 0.75$, so the characteristics of the tangential velocity above the impinging position, e.g., $r/R = 0.7$, have the opposite properties compared with those at $r/R = 0.8, 0.9, {\text{and 1}}.0$.

The distribution of the radial velocity (bottom left in Fig. 12) has a dent in the region around the circumferential position of $\varPsi = 270^{\circ}$ and two bulges on the two sides of the dent. This is mainly due to the induced velocity of a pair of vortices entering the propeller in the axial direction.

The profiles of the angle of attack of the blades at the aforementioned radial positions are presented in the bottom right of Fig. 12. As defined in Fig. 5, the angle of attack of the blade is determined by the axial velocity and the tangential velocity, so the radial velocity does not play a role here. The distribution of the angle of attack can be divided into three sections in the measured domain. The section on the left side (shown inside the red dashed rectangle) has a value that is higher than that on the right side (shown inside the blue-dashed rectangle). The section in the middle (shown inside the black-dashed rectangle) has the maximum value of the angle of attack.

In conclusion of this section, the PIV measurement results at the wall-perpendicular plane are analysed for a relatively highly loaded propeller with a low height ratio, i.e., $T_{c} = 42.1$ and $h/R = 1.46$. The velocity components in the polar coordinate system, as well as the angle of attack of the blade, are presented. The distributions of the velocity feature a pair of vortices entering the propeller at an oblique angle. The angle of attack is not uniform in the circumferential direction of the propeller due to the impingement of the vortex: near the symmetry line, i.e., $\varPsi = 270^{\circ}$, there is a pulse of angle of attack of the blade, and the angle of attack on the left-hand side of the measurement domain is also higher than that on the right-hand side.

- The effect of the thrust coefficient on the non-uniformity of the propeller inflow.

**Fig. 13:**
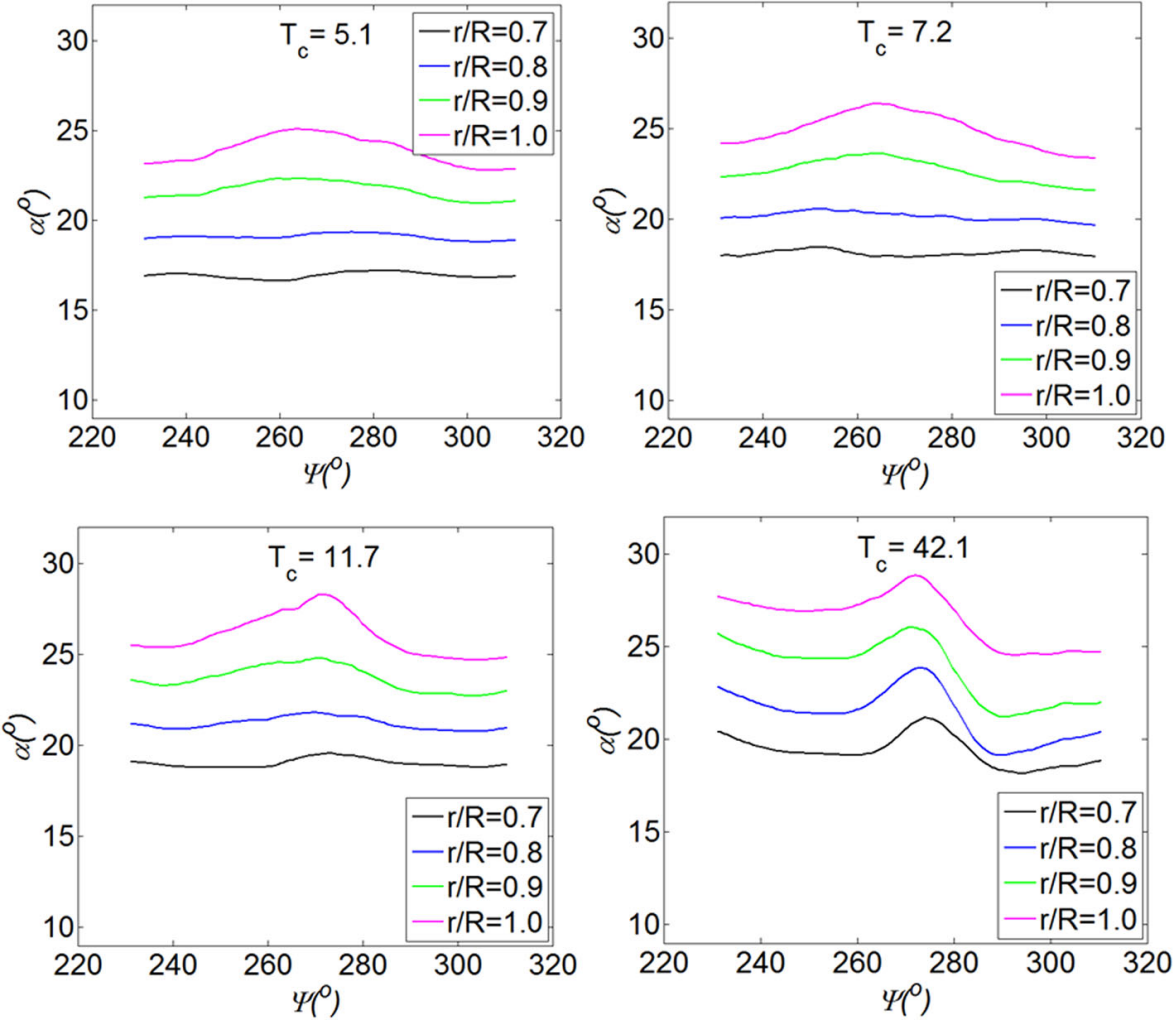
Angle of attack of the blade at different thrust coefficients of the propeller. *h/R* = 1.46

The distribution of the angle of attack at four different thrust coefficients is shown in Fig. 13. At each thrust coefficient, the angle of attack shows a bulge at the phase angle around $\varPsi = 270^{\circ }$. The ratio of the maximum angle of attack over the minimum ($\alpha_{ \hbox{max} } /\alpha_{ \hbox{min} }$) increases from 1.03 to 1.23 at radial position of $r/R = 0.9$ as the thrust coefficient increases from $T_{c} = 5.1$ to $T_{c} = 42.1$. As explained before, this bulge in the middle is due to the pair of vortices entering the propeller in the radial direction. At $T_{c} = 11.7$, the angle of attack on the left-hand side is slightly higher than that on the right-hand side; at $T_{c} = 42.1$, this step increases. As shown in the top right of Fig. 12, this step is due to the induced tangential velocity of the vortices that enter the propeller in the propeller axial direction.

From the above analysis, it is observed that for the cases with relatively low thrust coefficient, i.e., $T_{c} = 5.1, 7.2$, and 11.7, the flow field is featured by the induced velocity of the Z-component of the vorticity (in the radial direction of the propeller). As the thrust coefficient is high, i.e., $T_{c} = 42.1$, the flow field is influenced by both the *X* and *Z* components of the vorticity. This trend is further analysed by investigating the vortex trajectory at different thrust coefficients as below.

**Fig. 14:**
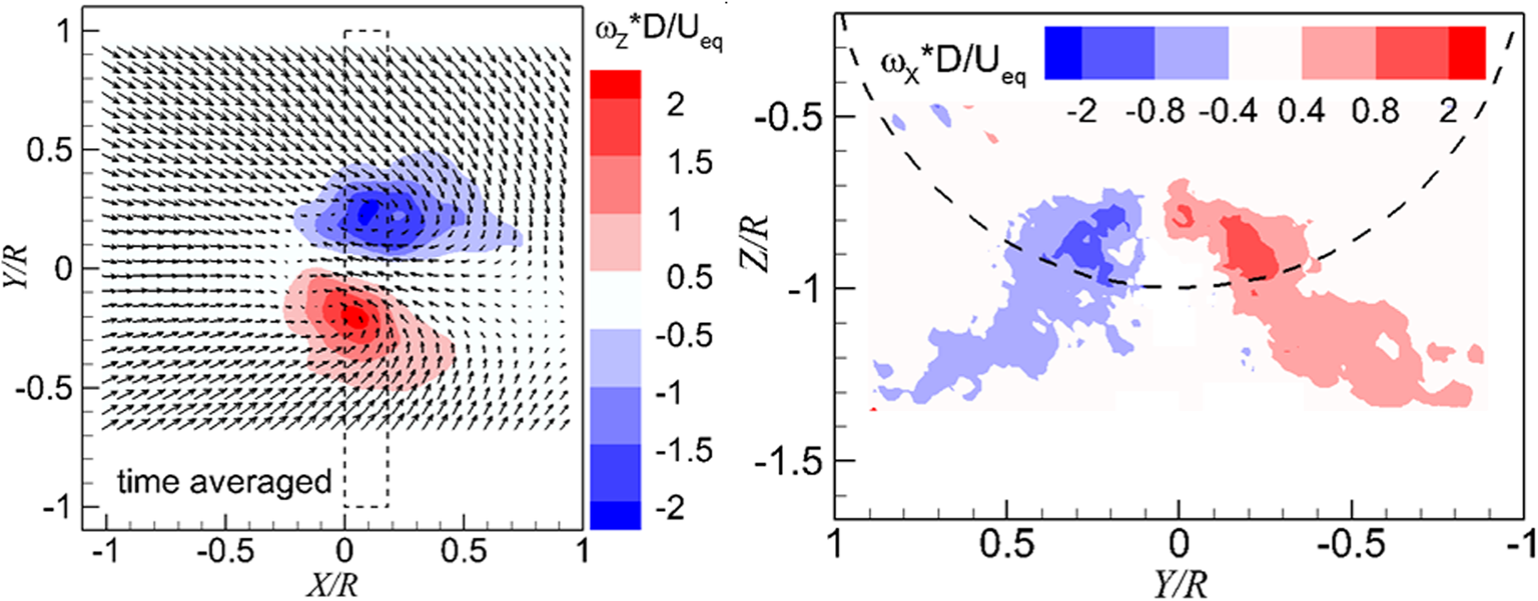
Time-averaged flow field. *Left* wall-parallel plane; *right* wall-normal plane directly upstream of the propeller. $T_{c}$  = *11.7*, *h/R* = 1.46

**Fig. 15:**
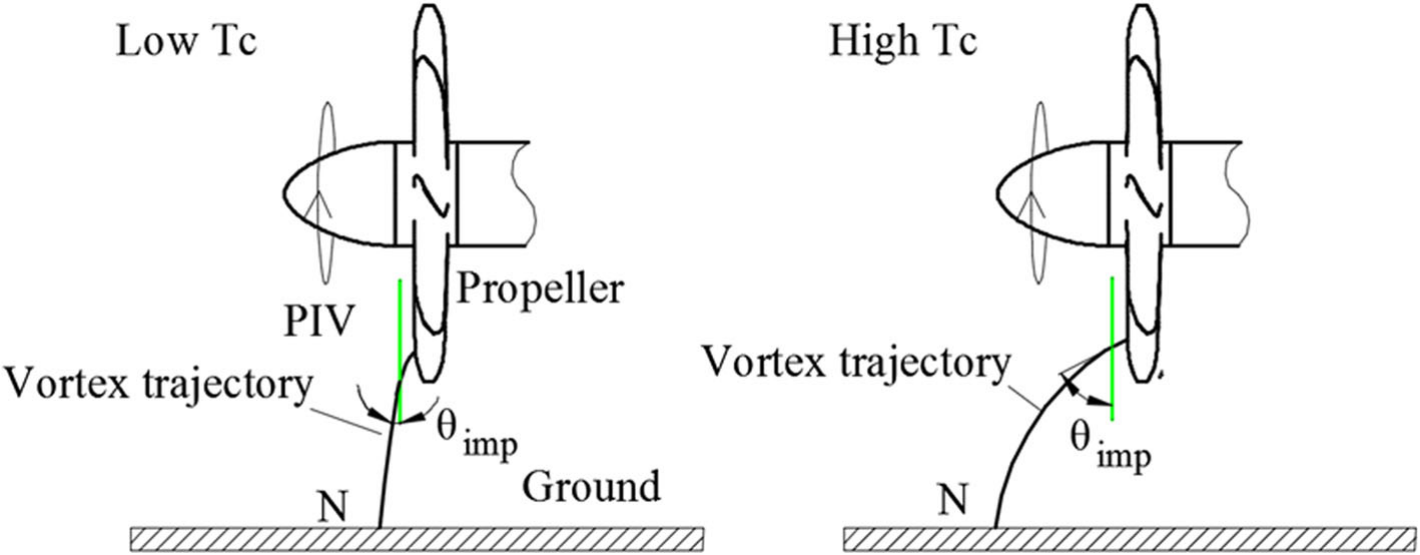
Schematic of the vortex trajectory at different loadings of the propeller (*side view*). ‘*N*’ is the ascending position of the ground vortex. $\theta_{\text{imp}}$ is the oblique angle of the trajectory of the impinging vortex relative to the PIV measurement plane (*green line*)

The time-averaged flow at $T_{c}$  = *11.7*, *h/R* = 1.46 is shown in Fig. 14. It is found that as the thrust coefficient decreases, the vortex foot moves downstream in the wall-parallel plane comparing with that as shown Fig. 8 [the same phenomenon was observed in Trapp and Girardi (2010)]. The entry position of the ground vortices into the propeller plane shows a negligible change. A schematic depicting this trend is shown in Fig. 15. The vortex trajectory at the PIV measurement plane has an oblique angle which is denoted as $\theta_{\text{imp}}$, and this angle increases as the thrust coefficient increases. This results in an increase of the axial component of the vorticity.

The foot of the ground vortex moving upstream as the thrust coefficient increases also implies that the occurrence of ground vortices is a local phenomenon. As the thrust coefficient increases, the stagnation point (line), i.e., the intersection point (line) between the stream tube of the propeller and the ground, moves downstream. Therefore, the ground vortices are not originated from these intersection positions that are mainly determined by the thrust coefficient of the propeller. Instead, the ground vortices moving upstream mean that there are locally formed stagnation points.

In conclusion for this section, it is shown that as the thrust coefficient of the propeller increases, the amount of vorticity entering the propeller stream tube and the resulted non-uniformity of the flow field increase. A similar research on a suction tube was reported in (Murphy and MacManus 2011a), and the distortion of the inflow of the suction tube shows an increase and then a decrease as the thrust coefficient [corresponding to the velocity ratio as defined in (Murphy and MacManus 2011a)] keeps increasing, which is different from our results. By checking the experimental setup in (Murphy and MacManus 2011a), it is found that the thrust coefficient is tuned by changing the velocity of the free stream. A higher thrust coefficient corresponds to a lower velocity of the free stream, which means a lower amount of vorticity in the far-field boundary layer. The vorticity in the far-field boundary layer is one source of ground vortices, in addition to the vorticity that is generated from the pressure gradient on the ground due to the propulsor suction effect. Therefore, there is a local maximum due these two opposing factors. However, the thrust coefficients in our tests are tuned by changing the rotating speed of the propeller whilst maintaining the free-stream velocity. The strength of the ground vortices keeps increasing as the thrust coefficient increases, so is the impact of ground vortices on the propeller inflow.

- The effect of the height ratio on the non-uniformity of the propeller inflow.

**Fig. 16:**
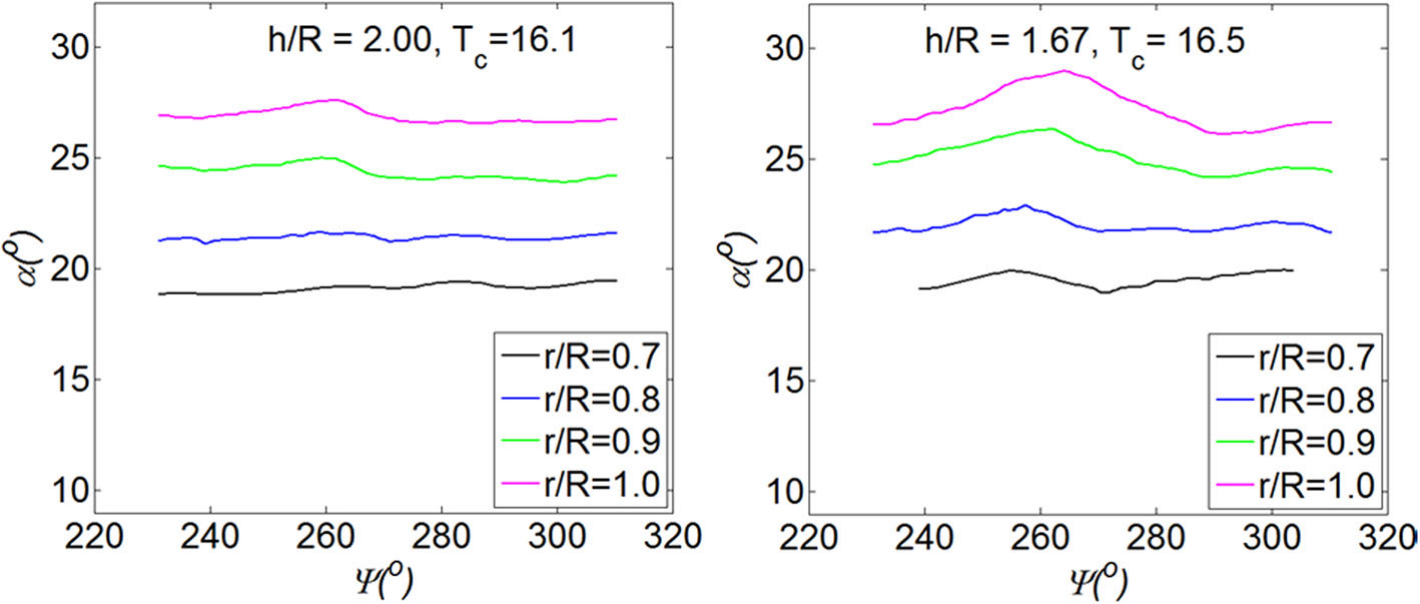
Angle of attack of the blade at different height ratios of the propeller

The angle of attack of the blade at different height ratios is shown in Fig. 16. At the height ratio of $h/R = 2.00$, the ratio of the angle of attack, $\alpha_{\hbox{max} } /\alpha_{\hbox{min} }$, is 1.05 at the radial position of ${\text{r}}/{\text{R}} = 0.9$; at the height ratio $h/R = 1.67$, the ratio of the angle of attack $\alpha_{\hbox{max} } /\alpha_{\hbox{min} }$ is 1.09. Therefore, as the height ratio decreases, the flow becomes more non-uniform as expected. This result is consistent with the result of a suction tube model which shows the strength of ground vortices increases as the height ratio decreases (Murphy and MacManus 2011b).

- Impact of ground vortices on the time-averaged performance of the propeller.

To determine the effect of ground vortices on the performance of the propeller, the data at two height ratios are compared, namely, $h/R = 3.0$ and $h/R = 1.46$. The height ratio of $h/R = 3.0$ is the maximum height ratio could be achieved in the setup. It is supposed that the strength of ground vortices generated at the height ratio of $h/R = 3.0$ is much smaller than that at $h/R = 1.46$. The height ratio of $h/R = 1.46$ is the position closest to the ground during our test, and it induces ground vortices which have the strongest impact on the propeller inflow.

**Fig. 17:**
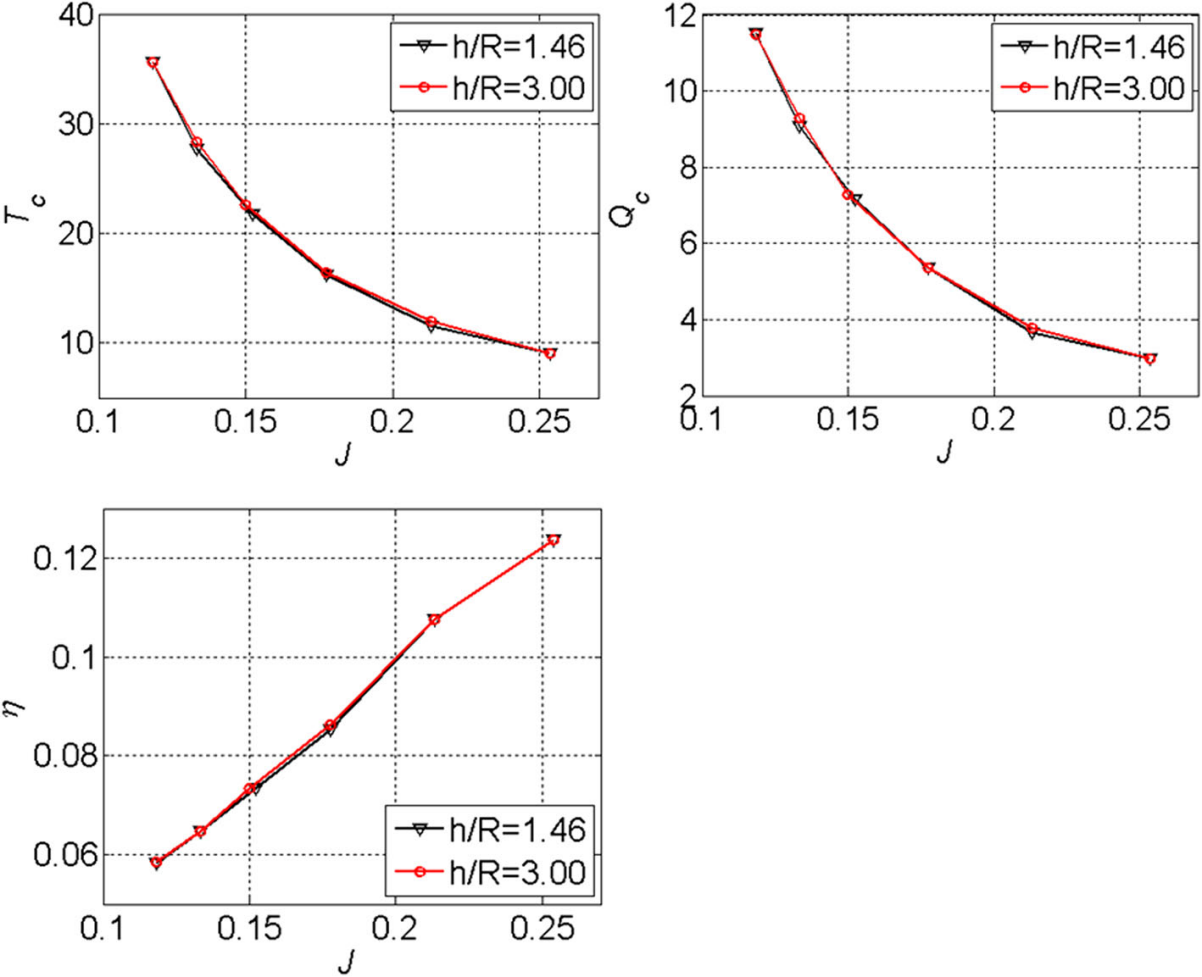
Impact of ground vortices on the time-averaged performance of the propeller. *Top left* thrust coefficient; *top right* torque coefficient; *bottom left* efficiency

The difference of the propeller performance between $h/R = 1.46$ and $h/R = 3.0$ is negligible, as shown in Fig. 17. This means that the time-averaged performance of the propeller is independent of the ground vortices. First, the effects of the vortices entering the propeller in the propeller axial direction are cancelled out by each other. This hypothesis is confirmed by the tangential velocity distribution, as shown in the top right of Fig. 12. Second, although the effect of vortices entering the propeller in the radial direction induces an axial velocity decrease in the propeller inflow (as shown in the top left of Fig. 12), this influenced region is small compared with the whole disk region of the propeller and its effect is negligible as well. As the majority of research on turbofans is conducted on suction tubes, the impact of ground vortices on the loadings of a turbofan is not available. Our tests on a propeller give such data for the first time.

## Conclusions

Particle image velocimetry measurements have been conducted to investigate the flow field generated by a propeller in ground proximity. At a low free-stream velocity, a highly loaded propeller in ground operation can induce vortical flow ascending from the ground to the propeller. A domain boundary of occurrence of ground vortices is built basing on the flow field near the ground and upstream of the propeller. As the distance between the propeller and the ground decreases, and as the thrust coefficient of the propeller increases, the occurrence of ground vortices is observed.

The vortices ascending from the ground enter the propeller plane at an oblique angle, where both the radial and axial (with respect to the propeller) components of the vorticity are present. The ground vortices entering the propeller at an oblique angle, as observed in our result, were not reported before in the studies on turbofans or suction tube models (De Siervi et al. 1982; Murphy and MacManus 2011a; Wang and Gursul 2012), because the shroud directs the inflow of the turbofan to be parallel with the axis of the engine. The relative strength of the two components (radial and axial) of the vorticity is dependent of the propeller thrust setting: the higher the thrust coefficient and the stronger the axial component of the vorticity. The axial component of the vorticity entering the propeller mainly influences the tangential and the radial components of the velocity of the propeller inflow; the radial component of the vorticity entering the propeller mainly influences the axial component of the velocity of the propeller inflow. Consequently, the blade incidence angle is changed and becomes non-uniform in the circumferential direction. With a higher thrust coefficient and a lower height ratio of the propeller, the non-uniformity of the blade incidence angle becomes more severe. The time-averaged performance of the propeller is independent of the ground vortices, because the effects of the vorticity cancel each other and the magnitude of the vorticity is relatively small.

## Abbreviations

FOD: Foreign object damage
OJF: Open jet facility
PIV: Particle Image Velocimetry
RSB: Rotating shaft balance
rpm: Revolutions per minute

## English symbols

$C_{\text{vort}}$: Concentration of vorticity
*D*: Diameter of the propeller
$h$: The distance between the propeller centre line and the ground
*J*: Advance ratio of the propeller
*n*: Rotating speed of the propeller (round per second)
*Q*: Torque of the propeller
$Q_{c}$: Torque coefficient of the propeller
*R*: Radius of the propeller
*Re*: Reynolds number
*T*: Thrust of the propeller
$T_{C}$: Thrust coefficient of the propeller
$U_{a0}$: The resultant axial velocity of the cross section of the blade
$U_{a,i}$: The induced axial velocity of the cross section of the blade
$U_{e}$: Effective velocity of the cross section of the blade
$U_{\text{eq}}$: Equivalent axial component of the velocity in the propeller inflow, determined from the actuator disk model
$U_{\text{in}}$: Inlet velocity of a turbofan or a suction tube
$U_{r}$: Measured radial component of the velocity in the propeller inflow
$U_{t}$: Measured tangential component of the velocity in the propeller inflow
$U_{t0}$: The resultant tangential velocity of the cross section of the blade
$U_{t,i}$: The induced tangential velocity of the cross section of the blade
$U_{\infty }$: Free-stream velocity
$U_{X}$: Measured axial component of the velocity in the propeller inflow
*X, Y, Z*: *X, Y,* and *Z* axes in the fixed coordinate system

## Greek symbols

$\alpha$: Angle of attack of the cross section of the blade
*β*: Geometry pitch angle of the cross section of the blade
$\delta_{l}$: Distance of the laser sheet from the reference position
$\eta$: Efficiency of the propeller
$\theta_{\text{imp}}$: Oblique angle of the trajectory of the impinging vortex
*ρ*: Density of the air
*ω*: Vorticity
$\left| \omega \right|_{\hbox{max} }$: Maximum vorticity magnitude in the time-averaged flow field
$\left| \omega \right|_{\text{unaff}}$: Vorticity magnitude in the unaffected region of ground vortices
